# Uniportal versus multiportal thoracoscopic lobectomy: Ergonomic evaluation and perioperative outcomes from a randomized and controlled trial

**DOI:** 10.1097/MD.0000000000022719

**Published:** 2020-10-16

**Authors:** Jie Yao, Zhibo Chang, Lin Zhu, Junqiang Fan

**Affiliations:** Department of Thoracic Surgery, Second Affiliated Hospital of Zhejiang University, School of Medicine, Hangzhou, China.

**Keywords:** ergonomic evaluation, lobectomy, lung cancer, uniportal, video-assisted thoracoscopic surgery

## Abstract

**Background:**

To compare perioperative outcomes and surgeon physical and mental stress when performing lobectomy through uniportal and multiportal video-assisted thoracoscopic surgery (VATS) on patients with non-small-cell lung cancer (NSCLC).

**Methods:**

Patients aged 41 to 73 years with resectable NSCLC were randomly assigned via a computer-generated randomisation sequence to receive either uniportal VATS (UVATS) or multiportal VATS (MVATS) lobectomy and lymphadenectomy between December 2015 and October 2016. Overall, we randomly assigned 35 patients to the UVATS and 34 to the MVATS group. Patients and the investigators undertaking interventions, assessing short-term outcomes, performing ergonomic evaluations, and analyzing data were not masked to group assignment.

**Results:**

Patient demographics of the 2 groups were comparable. The ergonomic evaluation considered eye blink rate and the NASA Task Load Index (NASA-TLX), better results were observed in UVATS than in MVATS. The operative time, number of lymph nodes harvested, chest tube duration, length of hospital stay, and lung function were not significantly different between the groups. Compared with MVATS lobectomy, UVATS lobectomy was associated with less intraoperative blood loss and less volume of total drainage in the 24 hours. No conversion, no reoperation, and no in-hospital mortality occurred in either group.

**Conclusions:**

UVATS lobectomy is a safe and programmable technique with some better perioperative outcomes and ergonomic results than MVATS. Further studies based on large numbers of patients and with long-term follow-up are required to confirm its benefits towards patients.

**Trial registration:**

ClinicalTrials.gov ID:NCT02462356. Registered May 27, 2015.

## 1 Introduction

With acquired experience and improved instruments, video-assisted thoracoscopic surgery (VATS) lobectomy has become widely used for early-stage non-small-cell lung cancer (NSCLC).^[1]^ Compared with thoracotomy, VATS is associated with reduced length of hospital stay, less postoperative pain, fewer postoperative complications, more rapid recovery to normal life, and less pulmonary injury without compromising oncology principles.^[2–4]^ However, such surgeries are conventionally multiportal (MVATS); the lobectomy for lung cancer can be accomplished with a single incision. Recently, uniportal thoracoscopic lobectomy has been accepted as a safe and effective surgical procedure for patients with lung cancer. Growing evidence indicates that the perioperative outcomes of UVATS are comparable with those of the MVATS approach, especially in terms of less access trauma and less intraoperative blood loss, and UVATS is being increasingly implemented worldwide.^[5]^ In addition, recent studies have shown that UVATS wedge resection may provide better ergonomics for the surgeon due to standing straight and facing the monitor with a more neutral body posture.^[6]^ Additionally, a previous review has suggested that ergonomic factors can influence surgical performance in laparoscopies.^[7]^ However, a significant limitation of most of these studies is their retrospective and descriptive design; no randomized trials have investigated the benefits of UVATS lobectomy.

Here, we compare uniportal with multiportal thoracoscopic lobectomy in patients with NSCLC to assess the short-term outcomes and surgeon ergonomics.

## 2 Methods

### 2.1 Patient selection

This prospective, randomized, controlled trial was registered with ClinicalTrials.gov (NCT02462356) and approved by the Ethics Committee of the Second Affiliated Hospital of Zhejiang University, School of Medicine. All patients provided written informed consent before operation. Clinical cancer stages were assessed during a preoperation examination including enhanced computed tomography, ultrasonography, echocardiography, brain magnetic resonance imaging, bronchoscope (if necessary), and bone emission computed tomography (as well as positron emission tomography-computed tomography, if possible).

The inclusion criteria for lobectomy were as follows: patients with clinical diagnosis of primary lung cancer; age between 35 and 75 years old; tumor size ≤5 cm, clinically staged T1-2N0-1M0, prepared for lobectomy and mediastinal lymph node dissection; American Society of Anesthesiology score of 0–1.

Exclusion criteria: Patients with N2- or N3-positive or distant metastasis; who had undergone neoadjuvant chemotherapy; with tumor invasion of peripheral structures; with previous history of thoracic operations; with serious thoracic adhesion; who will undergo pneumonectomy, sleeve lobectomy, segmentectomy, wedge resection; with preexisting chronic obstructive pulmonary disease, asthma, or interstitial lung disease; with cardiac, hepatic, or renal dysfunction.

To prevent surgeon bias, both UVATS and MVATS procedures were performed by Dr. Fan, who is experienced in both uniportal and multiportal VATS major pulmonary resection. Dr Fan began performing MVATS lobectomy in 2008 using a 3-incision approach, and performed his first UVATS lobectomy in December 2013. In the time since, >2000 cases of UVATS procedures have been carried out in our center.

Clinical features were recorded for all patients, including demographics, forced expiratory volume in the first second (FEV_1_), FEV_1_%, tumor location and diameter, tumor stage, histology, surgical time, estimated volume of blood loss, perioperative complications, chest tube duration, length of hospital stay, number of lymph nodes harvested, total drainage in the first 24 hours, and mortality. We used a computer-generated randomization sequence to randomly assign patients at a 1:1 ratio to undergo either uniportal or multiportal VATS lobectomy.

### 2.2 Anesthesia and analgesia

All patients received general anesthesia and were provided with patient-controlled analgesia. During operation, patients were placed in the full lateral decubitus position with single lung ventilation using a double-lumen endotracheal tube. All patients were extubated at the end of the procedure in the postanesthesia intensive care unit and transferred to the thoracic ward.

### 2.3 Operative procedure

#### 2.3.1 Thoracoscopic lobectomy via UVATS

**Figure 1:**
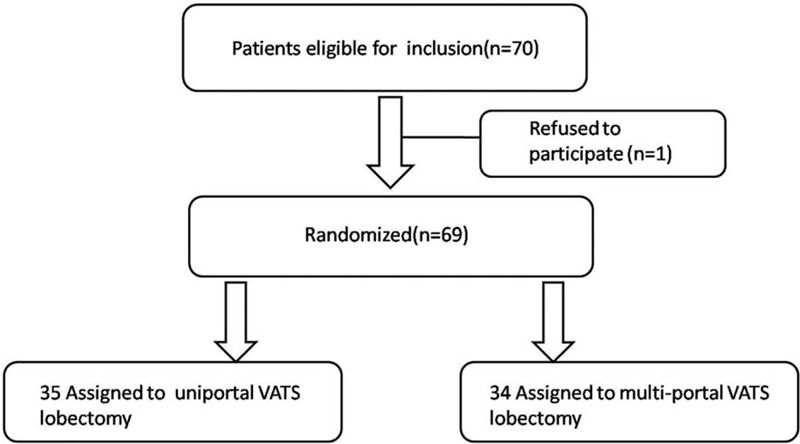
Flow chart of the study. VATS = video-assissted thoracic surgery.

The surgical instruments used were designed for UVATS, with double articulation and long curved suction (Fig. 1A and B). We prefer to create an incision about 3.5 cm in length at the fifth intercostal space, between the anterior axillary line and posterior axillary line (Fig. 1C). A 30°, 10-mm high definition camera thoracoscope was used to provide a panoramic view and placed at the posterior part of the incision during most of the operative time (to relieve fatigue, a transfusion tube binds the thoracoscope fixed by a vessel clamp^[8,9]^) (Fig. 1D). Both the surgeon and assistant who maneuvered the thoracoscope stood at the anterior side of the patient in order to have the same perspective during operation; when dissecting lymph nodes, a second assistant who retracted the lung to a better exposure stood on the back side.

A small disposable, plastic wound protector was used to stretch open the incision. This improved the camera's angle of vision and diminished compression of the intercostal space. UVATS lobectomy for lower lobes presented less difficulty, and were performed similar to conventional 3-port VATS as follows: dissection of the fissure, pulmonary artery, inferior pulmonary ligament, pulmonary vein, and bronchus. When the fissure was not complete, we used fissureless techniques, which procedure may be performed from bottom to top, the sequence for dissection being as follows: inferior ligament, inferior pulmonary vein, bronchus, pulmonary artery, and finally fissure stapling. For the upper lobes, we prefer to divide the anterior and apical arteries first in order to facilitate division of the upper lobe vein. The vessels were usually divided by staplers as recommended; when the angle for vascular division made stapler insertion difficult, a hem-o-lok or suture ligation was usually used, with suture ligation preferred (because a hemolock slips away easily).

**Figure 2:**
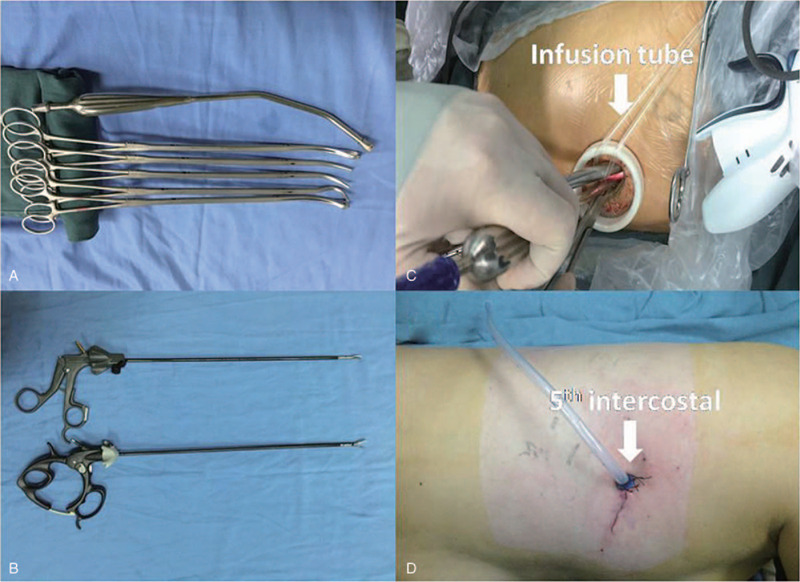
A and B: The designed surgical instruments with double articulate and long curve suction for uniportal video-assisted thoracoscopic surgery. C: A 30°, 10-mm high definition camera thoracoscope was placed at the posterior part of the incision during most of operative time (a transfusion tube binds the thoracoscope fixed by a clamp for relieving the tiredness), and other instruments were placed at the anterior side. D: A 3.5 cm incision was made at the fifth intercostal space that between the anterior axillary line and posterior axillary line, and a 24F chest tube was placed at posterior part of incision.

Systematic mediastinal lymphadenectomy can be performed according to oncologic criteria with similar results as conventional MVATS. For dissection of lymph node stations 2 and 4 (Fig. 2A), we recommend to lift the azygos vein and pull the superior vena cava to the right side by curve suction. This technique allows us to dissect the paratracheal lymph node easily. The station 4 lymph nodes in the left side were dissected using a combination of blunt and sharp techniques (Fig. 2D), with special care for exposure and to preserve the left recurrent laryngeal nerve. The station 5 and 6 lymph nodes were dissected with protection of the phrenic and vagus nerves. However, dissection of the left subcarnial lymph nodes was challenging. On the left side (Fig. 2C), right-sided double-lumen tube intubated may facilitate exposure; we retracted the left lower lobe anterior and pushed the esophagus posterior using lymph node clamps. A clearer visual of the subcarinal lymphatic tissue was thus achieved, making dissection easier. On the right side (Fig. 2B), we retracted the lobe with forceps and protected the esophagus with lymph node clamps and suction, thereby exposing the subcarinal area.

After the lobe was removed and placed in a protective bag, a systematic lymph node dissection was performed. A 24-F chest tube was placed in the posterior part of the incision; this chest tube was removed when there was no air leakage, no chylothorax, and the volume of drainage was <200 mL per day.

#### 2.3.2 Thoracoscopic lobectomy via MVATS

The initial camera port (1.0-cm-long incision) was made in the 7th intercostal space in the midaxillary line; the utility incision (4 cm long) was placed at the level of the 4th intercostal space in the anterior axillary line. An additional 0.5-cm-long insicion was performed in the 7th intercostal space in the posterior axillary line. As described in a previous study,^[10]^ the pulmonary vessels and bronchus were dissected using endoscopic staplers. A systemic mediastinal lymphadenectomy was performed the same way as for UVATS. A 24-F chest tube was placed through the observational incision; this chest tube was removed when there was no air leakage, no chylothorax, and the volume of drainage was <200 mL per day.

### 2.4 Ergonomic evaluation

During the operation, the surgeon's eye motions were captured and video was recorded as previously described.^[11]^ The video was recorded by an observer using an iPhone Plus 6 (Apple Inc., Cupertino, CA) with capture every 5 minutes and lasting for 1 minute. After the operation, blink data were processed from the recorded video. Blinks per minute were determined at the beginning (R1) and at the end (R2) of the operation, and the difference in blink rate as R1–R2. Upon completion of the surgery, the surgeon filled out the NASA-TLX form to evaluate the workload experienced. Specifically, the surgeon was asked to record his perceived mental, physical, and time demands on a 20-point scale (range 0–100), as well as his effort, performance, and frustration during the operation. The NASA-TLX has been used extensively in a variety of projects for assessing the perception of mental workload. The data collected were compared between the 2 VATS lobectomy approaches.

### 2.5 Statistical analysis

We used a desired power and precision to calculate the necessary sample size. Data were expressed as median and range for continuous variables, or mean and SD when appropriate. We compared groups with an independent samples *t* test when appropriate, and otherwise a Mann–Whitney *U* test or Chi-squared test. Statistical analyses were performed with SPSS (version 17) (spss, IBM, Chicago, IL, USA).

## 3 Results

**Figure 3:**
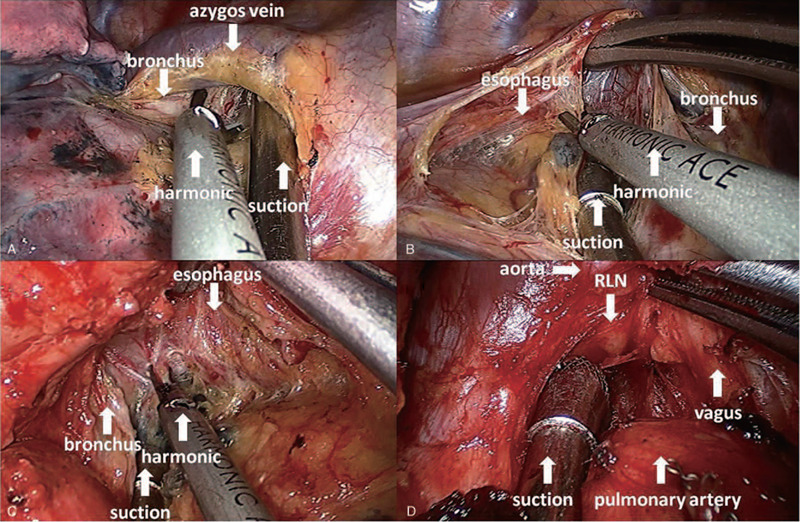
The surgeon with harmonic in right hand and suction in left hand in the mediastinal lymph nodes dissection period. A: 2R and 4R lymph nodes dissection. B: 7R lymph nodes dissection. C: 7L lymph nodes dissection. D: 4L lymph nodes dissection.

Figure 3 shows the study profile. Patients were recruited from December 2015 through October 2016, and 69 of 70 consecutive patients were randomly assigned to receive UVATS or MVTAS at the Second Affiliated Hospital of Zhejiang University, School of Medicine (1 refused to participate). After enrollment and randomization, 35 patients were analyzed in the UVATS group and 34 patients in the MVATS group. The demographic and clinical characteristics of the 2 groups were similar at baseline (Table 1). All UVATS patients underwent surgery without conversion to open thoracotomy or multiportal VATS, and there were no conversions from MVATS to open thoracotomy.

**Table 1:**
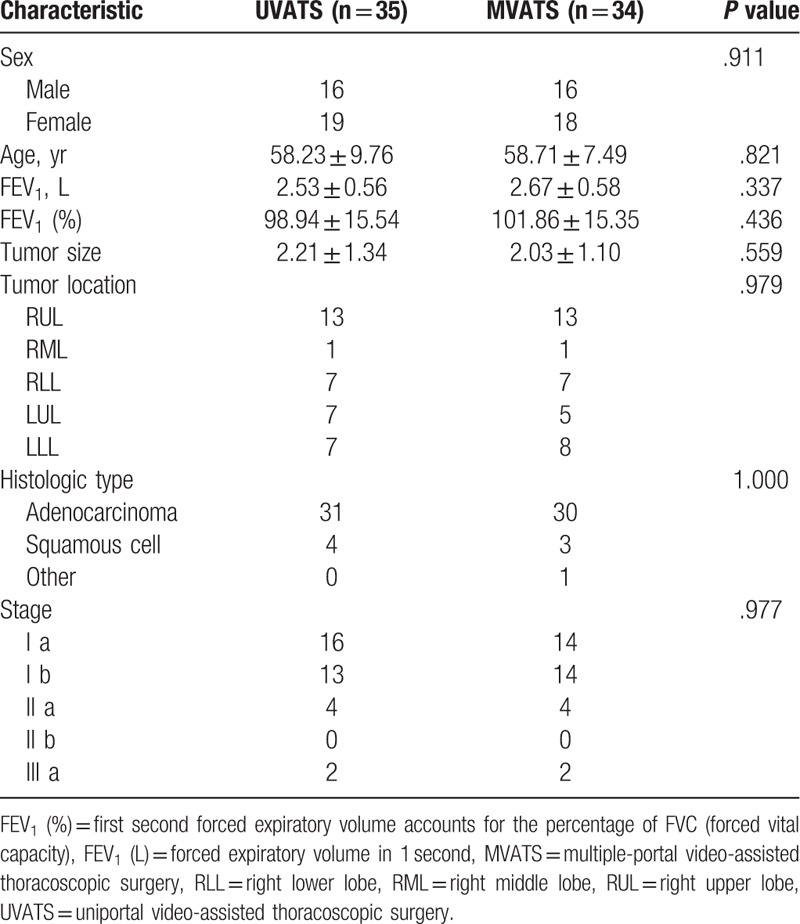
Patient demographics.

There were no significant differences in baseline characteristics between the UVATS and MVATS groups. The mean follow up was 3 months for both groups, and all completed the final survey. No patient deaths occurred during the follow-up period. Complications were observed in 13 patients (11.4% in UVATS vs 26.5% in MVATS groups). Although the surgical time, numbers of lymph nodes harvested, length of hospital stay, and chest tube duration were similar between both groups, less intraoperative blood loss (35.1 ± 25.0 vs 51.5 ± 40.8, *P* = .048) and less volume of total drainage in the first 24 hours (230.8 ± 117.2 vs 308.2 ± 145.1, *P* = .018) were observed for patients undergoing UVATS compared with MVATS.

A descriptive analysis of surgeon blink rate and NASA-TLX responses are reported in Table 2. At the beginning of the operation, blink rates (R1) in the UVATS and MVATS groups were comparable (8.31 ± 2.29 vs 8.44 ± 2.44, *P* = .981). However, the concluding blink rate (R2) was lower in the MVATS group (4.09 ± 1.68 vs 5.46 ± 2.02, *P* = .007), and the difference in blink rate (R1–R2) was significantly greater in the MVATS group (4.32 ± 2.63 vs 2.86 ± 1.38, *P* = .02). A less frequent blink rate was associated with a high effort level (45.3 ± 3.94 vs 38.86 ± 4.55, *P* < .001), greater mental demand (43.33 ± 4.27 vs 32.14 ± 5.46, *P* < .001), greater physical demand (44.70 ± 4.13 vs 29.86 ± 5.88, *P* < .001), and feeling more frustration (37.73 ± 3.56 vs 34.29 ± 4.56, *P* = .002).

**Table 2:**
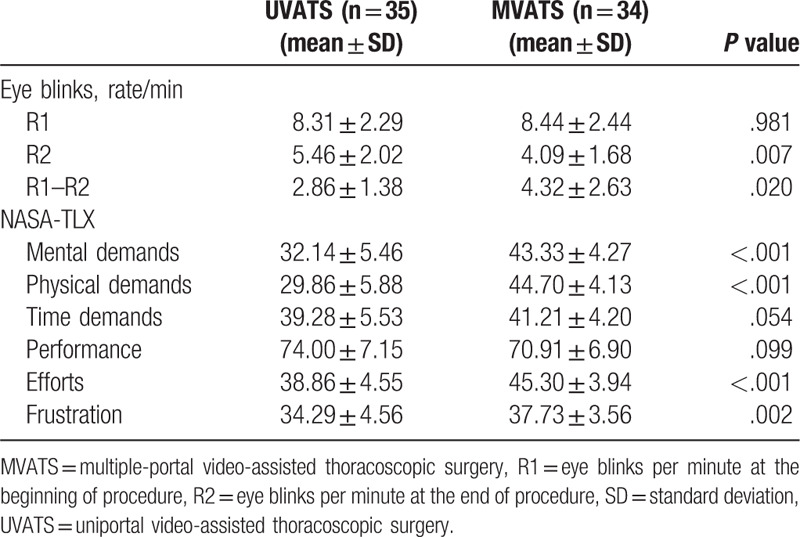
Ergonomic evaluation.

A total of 13 patients developed complications (4 vs 9 in UVATS and MVATS groups, *P* = .276). The rates of common complications after VATS lobectomy were similar in both groups—prolonged air leak lasting for >6 days (0 in UVATS vs 4 in MVATS), chylothorax (0 in UVATS vs 1 in MVATS), reinsertion of chest tube (4 in UVATS vs 3 in MVATS), and hoarseness (0 in UVATS vs 1 in MVATS). Details regarding complications are summarized in Table 3.

**Table 3:**
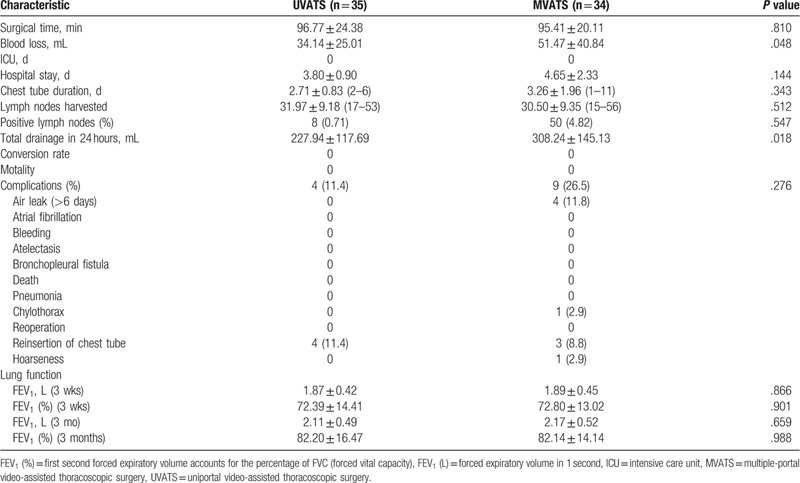
Surgical and postoperative data.

## 4 Discussion

In this study, we compared UVATS and MVATS lobectomy for NSCLC. UVATS procedure resulted in less total drainage in the first 24 hours, better ergonomics, and less intraoperative blood loss, without compromising safety or oncology principles. However, our findings demonstrated that there were no significant differences between the 2 treatment groups in regard to the number of lymph nodes harvested, surgical time, length of hospital stay, chest tube duration, or rate of conversion to open thoracotomy. Thus, UVATS lobectomy should be considered for this difficult and demanding operation.

Rocco et al^[12]^ first described UVATS pulmonary resections in 2004. Since then, UVATS has been performed for diagnostic and therapeutic procedures, such as pleural and mediastinal biopsies, deloculation of pleural effusion, pleurodesis, and lung wedge resection. In 2010, Gonzalez Rivas group reported the world's first UVATS lobectomy, which was a historic milestone for this uniportal approach.^[13]^ More recently, UVATS development has grown in popularity, and complex UVATS major lung resections have been performed (involving pneumonectomy, segmentectomy, bronchoplasty, and sleeve lobectomy).^[14–16]^ We have previously presented preliminary results of using UVATS lobectomy for locally advanced lung cancer.^[9]^ However, extremely few reports exist comparing UVATS and MVATS lobectomy with radical mediastinal lymph node dissection for NSCLC.

Evidence has shown that patients benefit from VATS being a less traumatic procedure compared than thoracotomy.^[17]^ However, controversy exists regarding the optimal number of ports for thoracoscopic lobectomy, with advantages and disadvantages being reported for both UVATS and MVATS lobectomy. However, these published studies were from non-randomized series, and the conclusions obtained might have been affected by differences in surgeons’ previous experiences and by selection bias. Our research directly compared UVATS and MVATS, and Dr Fan's experience with both approaches was sufficient to bypass the learning curve plateau. In addition, to reduce the chance for technical bias due to improvements in VATS during the course of the study, all patients were enrolled over a relatively short interval of 7 months and from a single center.

Our results showed less intraoperative blood loss, less volume of total drainage in the first 24 hours for the UVATS group. Personally, and admittedly naively, I would expect the “single portal versus multiple portal” explanation probably due to the experience and good cooperation of the surgical team. The shorter hospital stay observed for the UVATS group in our trial was not significant, which may not only indicate a faster postoperative recovery than from MVATS, but also a reduced cost of hospitalization. The median length of hospital stay in our study was 3.8 days, even less than previously reported.^[18,19]^

With respect to postoperative complications, there were no differences between the UVATS and MVATS group, and no conversion to thoracotomy was recorded in either group. This suggests that both access approaches are safe and feasible for VATS lobectomy. However, the UVATS approach was ergonomically superior to MVATS.

We recorded surgeon eye blink rates and self-reported NASA-TLX responses as markers of mental workload. Decreased blink rate has been reported to correlate with increased mental workload.^[20]^ We found that the surgeon's blink rate was reduced more for the UVATS group than for the MVATS group. Similarly, mental demands, physical demands, frustration, and effort were significantly lower after performing UVATS than after MVATS. These findings indicate that significant ergonomic benefits might be gained through using UVATS, which could be explained by the following: Firstly, in our implementation of UVATS, the surgeon and the assistant were placed in front of the patient so that they had the same perspective, improving coordination. Secondly, the UVATS procedure can improve body posture during surgery because the surgeon and assistant can stand straight and face the monitor with minimal neck movement. The more neutral body posture enables manipulation without influencing instrument movement. Thirdly, viewing direction vision was the same as the open thoracotomy, which reduces the depth of intraoperative visualization.

There remain several limitations of this study. First, the clinical trial involved only one experienced surgeon at a single center, and the results cannot be generalized to clinical settings. Second, a relatively small sample size was used in this study. Third, the ergonomic evaluation was conducted as an unblinded study.

## 5 Conclusion

Although our findings suggest that UVATS is more beneficial than MVATS in the treatment of NSCLC, it might not be possible to apply our findings to centers with less experience in UVATS procedures. We believe further studies should be done to compare UVATS with MVATS in a large number of patients, and preferably in multiple center settings that include both large-volume and small-volume centers. In addition, long-term outcome measurements comparing UVATS with MVATS are planned for the future, including overall survival analyses.

## Author contributions

**Conceptualization:** Junqiang Fan.

**Data curation:** Jie yao, Lin Zhu.

**Funding acquisition:** Jie yao.

**Investigation:** Zhibo Chang.

**Methodology:** Lin Zhu.

**Project administration:** Zhibo Chang.

**Resources:** Lin Zhu, Junqiang Fan.

**Writing – original draft:** Jie yao.

**Writing – review & editing:** Jie yao.
